# Genome‐Wide Screening in Haploid Stem Cells Reveals Synthetic Lethality Targeting *MLH1* and *TP53* Deficient Tumours

**DOI:** 10.1111/cpr.13788

**Published:** 2025-01-15

**Authors:** Rivki Cashman, Guy Haim‐Abadi, Elyad Lezmi, Hagit Philip, Jonathan Nissenbaum, Ruth Viner‐Breuer, Chen Kozulin, Tamar Golan‐Lev, Aseel Gadban, Shiri Spinner‐Potesky, Ofra Yanuka, Oded Kopper, Nissim Benvenisty

**Affiliations:** ^1^ NewStem LTD Jerusalem Israel; ^2^ The Azrieli Center for Stem Cells and Genetic Research, Department of Genetics, Silberman Institute of Life Sciences The Hebrew University Jerusalem Israel

## Abstract

Synthetic lethality is defined as a type of genetic interaction where the combination of two genetic events results in cell death, whereas each of them separately does not. Synthetic lethality can be a useful tool in personalised oncology. *MLH1* is a cancer‐related gene that has a central role in DNA mismatch‐repair and *TP53* is the most frequently mutated gene in cancer. To identify genetic events that can lead to tumour death once either *MLH1* or *TP53* is mutated, a genome‐wide genetic screening was performed. Thus, mutations in all protein‐coding genes were introduced into haploid human embryonic stem cells (hESCs) with and without loss‐of‐function mutations in the *MLH1* or *TP53* genes. These experiments uncovered a list of putative hits with *EXO1*, *NR5A2*, and *PLK2* genes for *MLH1,* and *MYH10* gene for *TP53* emerging as the most promising candidates. Synthetic lethal interactions of these genes were validated genetically or chemically using small molecules that inhibit these genes. The specific effects of SR1848, which inhibits NR5A2, ON1231320 or BI2536, which inhibits PLK2, and blebbistatin, which inhibits MYH10, were further validated in cancer cell lines. Finally, animal studies with CCL xenografts showed the selective effect of the small molecule BI2536 on *MLH1*‐null tumours and of blebbistatin on *TP53‐*mutated tumours. Thus, demonstrating their potential for personalised medicine, and the robustness of genetic screening in haploid hESCs in the context of cancer therapeutics.

## Graphical abstract

To identify genetic events that can lead to tumour death once either *MLH1* or *TP53* is mutated, a genome‐wide genetic screening was performed, uncovering a list of putative hits. Synthetic lethal interactions were validated either genetically or chemically by using small molecules that inhibit these genes either in vitro and in vivo.

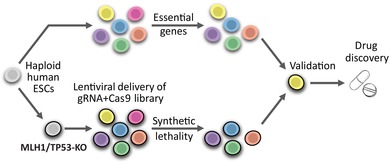

## 1 Introduction

In the relentless pursuit of effective cancer therapies, scientists and researchers have embraced innovative strategies to target cancer cells selectively. One such promising approach is synthetic lethality (SL)‐ a concept that holds great potential in the development of novel drugs for cancer treatment [1, 2, 3]. SL refers to the concept of exploiting the genetic dependencies that arise when two or more genes or pathways function synergistically to maintain cellular survival. Disrupting one of these genes or pathways may not be lethal on its own, but in the presence of an existing genetic defect or alteration, it becomes synthetically lethal, resulting in cell death. SL exploits the genetic vulnerabilities present in cancer cells, offering a unique avenue to selectively eradicate these cells while sparing normal, healthy cells. This targeted strategy offers a promising alternative to traditional cancer treatments by capitalising on the specific genetic alterations found in cancer cells [2, 4, 5].

Despite the immense potential of SL, only a single family of drugs, the poly (ADP‐ribose) polymerase (PARP) inhibitors, employing this groundbreaking approach in patients carrying BRCA mutations has made its way into full clinical use [6]. This underscores both the complexity of translating SL concepts into effective therapeutic strategies and the need for further research to unlock its full potential in cancer treatment.

Cancer cell lines (CCLs) have become indispensable tools in the realm of cancer research and genome‐wide screening utilising this platform has emerged as a powerful technique, allowing the identification of potential SL interactions [7, 8]. However, despite its utility, this approach is not without its limitations. One of these limitations is the inherent polyploidy of CCLs. In an attempt to overcome this drawback, a unique near haploid CCL, KBM7 and its derivative HAP1, were used in a genome‐wide genetic screen to establish an SL interaction network [9]. Nevertheless, all CCLs carry multiple mutations including in central tumour suppressor genes such as *TP53*, thus limiting their utility.

Genome‐wide screening using haploid human embryonic stem cells (hESCs) has proven as a powerful tool for the identification of essential genes and the uncovering of genes and pathways that play an important role in the differentiation into the three embryonic germ layers [10, 11]. Their unique characteristics, which include fast cell division while maintaining genomic integrity and one copy of the genome that increases the robustness of a genome‐wide screening make them the superior tool to overcome the inherent complexities of CCLs in the quest for SL targets [12, 13].

To exploit haploid hESC potential to uncover novel SL interactions, we chose the *MLH1* and *TP53* genes that play important roles in cancer. *MLH1* is a cancer‐related gene that has a central role in DNA mismatch repair (MMR) [14, 15, 16]. MMR deficiency leads to high microsatellite instability (MSI‐H) and the accumulation of mutations across the genome [17, 18, 19]. Deficiency in the MMR pathway is common in colorectal cancer (~15%) [20], which is the fourth most common cancer and the second most common cause of cancer‐related deaths, as well as additional types of gastrointestinal cancer, and can also be found in the uterus, breast, prostate, bladder, and thyroid cancers [21, 22, 23, 24, 25]. *TP53* is the most frequently mutated gene in cancer [26, 27], and in response to DNA damage, initiates cell cycle arrest to allow either DNA repair or apoptosis [28]. Complete loss‐of‐function mutations that lead to p53 protein loss occur in a high percentage of colorectal cancer [29, 30, 31].

We utilised haploid hESCs to develop models for the loss of either *MLH1* or *TP53* genes and demonstrated the biological relevance of these models. Next, we used these models for genome‐wide loss‐of‐function CRISPR screens and identified novel potential SL interaction targets. We thus examined the SL interactions of *EXO1*, *NR5A2*, and *PLK2* genes with *MLH1*, and of *MYH10* gene with *TP53*. Through comprehensive genetic and chemical validations both in vitro and in vivo, we validated the SL of these genes with *MLH1* and *TP53*.

## 2 Results

### 2.1 Establishing and Validating Models for Loss of MLH1 and p53 in Haploid hESCs

**FIGURE 1:**
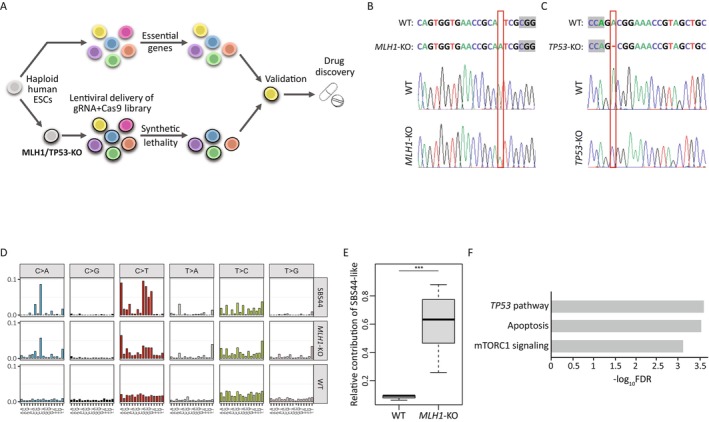
Establishing and validating haploid models for synthetic lethality genome‐wide screening. (A). Schematic illustration of the experiment. (B‐C). Sanger sequencing results of *MLH1‐*KO (B) *and TP53‐*KO (C) demonstrate one nucleotide insertion (B) or deletion (C) that causes a frameshift. The PAM sequence is highlighted in grey and mutation loci are in red rectangles. (D). The similarity between the SBS44 signature—associated with defective DNA mismatch repair (upper panel) to the *MLH1*‐KO (middle panel) and WT clones (lower panel). The colours describe the different types of base changes. The x‐axis is all possible single‐base‐substitutions (SBS), and the y‐axis is the frequency of each substitution in the different groups. (E). Relative contribution of SBS44‐like signature to WT or *MLH1*‐KO cells. Shown are box plot representations, with the median as the centre line, and 25% and 75% as box limits, *n* = 3 and 8 for wild type and *MLH1*‐KO clones that were analysed utilising whole‐genome sequencing, respectively. ***p < 0.001, by two‐sided *t*‐test. (F). Gene set enrichment analysis (GSEA) was performed on the RNA‐seq results comparing *TP53*‐KO cells to WT cells demonstrating enrichment of *TP53*‐related pathways.

This study presents the development of an innovative pipeline that utilises haploid hESCs to identify SL interactions and facilitate drug development (Figure 1A). The first step in this process required establishing haploid hESC models for cancer. In this research, models for MMR and p53 deficient tumours have been established. Thus, haploid hESCs were transiently transfected with a vector containing GFP, Cas9, and single guide RNAs (sgRNAs) for either *MLH1* or *TP53* genes. Clones were isolated from transfected cells and the introduction of a loss‐of‐function mutation to the *MLH1* and *TP53* genes was validated with Sanger sequencing and western‐blot analysis (Figure 1B,C, and Figure S1A,B).

Tumour cells are known to accumulate mutations that can be classified into different signatures. These mutation signatures are the result of various biological perturbations and mutational processes that occur during the disease. To demonstrate the biological relevance of the haploid *MLH1*‐knock‐out (KO) hESCs, their mutational signatures were examined. Thus, *MLH1* mutated haploid hESCs were grown for 3 months to allow mutation accumulation and subclones of *MLH1*‐KO (*n* = 8) and wild‐type (WT) (*n* = 3) cells were established and whole‐genome sequencing analysis was performed. Using the Cosmic sigProfiler (https://cancer.sanger.ac.uk/signatures/tools/), two signatures, SBS44 and ID2, which correlate with MMR deficiency were identified (Figure 1D and Figure S1C, respectively). These signatures were identified in *MLH1*‐KO and not in the WT cells (Figure 1E and Figure S1D, respectively) and are highly represented in MMR‐deficient tumours, such as the uterus and colorectal cancers. Thus, demonstrating that the haploid hESC MMR model recapitulates the mutational process observed in patients with MMR deficiency.

To highlight the biological significance of the haploid *TP53*‐KO hESCs, a comparison of gene expression was conducted between the WT and *TP53*‐KO hESCs. Gene set enrichment analysis (GSEA) of the most downregulated genes in *TP53*‐KO cells showed enrichment for pathways related to *TP53*, such as the *TP53* pathway, apoptosis, and mTORC1 signalling (Figure 1F).

### 2.2 Genome‐Wide and Custom‐Made Genetic Screening for SL Interaction Identification

**FIGURE 2:**
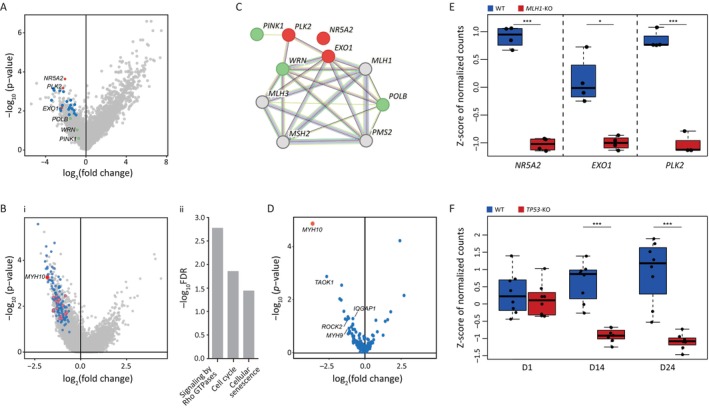
Genome‐wide and custom‐made genetic screening for synthetic lethality interaction identification. (A). Volcano plot displaying the CRISPR score (CS = log_2_ (fold change) of *MLH1*‐KO vs. WT) and adjusted *p*‐values for all the nuclear genes in the genome‐wide screen. Blue dots = depleted genes that were selected for further analysis. Red dots = three most promising candidate genes. Green dots = three genes previously suggested as synthetic lethal partners for mismatch repair (MMR) deficiency. (B) (i): Volcano plot displaying the CRISPR score (CS = log_2_ (fold change) of *TP53*‐KO vs. WT) and adjusted *p*‐values for all the genes in the genome‐wide screen. Blue dots = significantly depleted genes that were selected for further analysis. Light red dots = genes related to signalling by Rho GTPases. Red dot = *MYH10*. (ii): Gene set enrichment analysis (GSEA) was performed on the significantly depleted genes that were highlighted in B‐i. (C). STRING analysis results indicate the connection between MMR genes (grey) and the genes highlighted in red/green on the volcano plot (A). (D). Volcano plot displaying the CRISPR score (CS = log_2_(fold change) of *TP53*‐KO vs. WT) and adjusted *p*‐values for the genes in the costume screen. (E). Boxplot of *NR5A2* (left), *EXO1* (middle), and *PLK2* (right) sgRNAs distribution in the different samples = WT (blue) /*MLH1*‐KO (red), on the last time point. Read counts were normalised by each sample (read count per sgRNA/total counts in a sample), and then the Z‐score was calculated for each sgRNA across all samples. Shown are box plot representations, with the median as centre line, and 25% and 75% as box limits, *n* = 4. *p < 0.05, ***p < 0.001, by two‐sided *t*‐test. (**F**). Boxplot of *MYH10* sgRNAs distribution in the different samples—WT (blue) /*TP53*‐KO (red), and time points (x‐axis). Read counts were normalised by each sample (read count per sgRNA/total counts in a sample) and then the *Z*‐score was calculated for each sgRNA across all samples. Shown are box plot representations, with the median as the centre line, and 25% and 75% as box limits, *n* = 8. ***p < 0.001, by two‐sided *t*‐test.

Following the construction and validation of the cancer models, we embarked on genome‐wide CRISPR‐Cas9 screens to uncover novel SL interactions with either the *MLH1* or *TP53* genes. Thus, WT, *MLH1*‐KO, and *TP53*‐KO haploid hESCs were transduced with the Brunello LentiCRISPR knockout pooled library that targets approximately 19,000 genes, through ~76,400 unique sgRNAs [32]. Genomic DNA was extracted at different time points, and libraries for next‐generation sequencing (NGS) were prepared using distinct primers designed to identify the pooled sgRNAs. To identify genes that were specifically depleted in either the *MLH1* or *TP53* screens and not in the WT screen, the final time points of the three screens were compared. This analysis yielded a CRISPR score (CS) and an adjusted *p*‐value for each gene (Figure 2A,B). To identify the most promising candidate genes for SL interactions, the following selection criteria were applied: (1) Top‐ranked depleted CS and *p*‐value in the different comparison groups; (2) gene expression in hESCs above a defined threshold, and (3) a significant separation in sgRNA distribution between mutated and WT samples (for more details see relevant section in Material and Methods). Guided by these criteria, lists of 49 genes for the *MLH1*‐KO model and 132 genes for the *TP53*‐KO model were generated (Figure 2A,B‐i, respectively).

We prioritised genes that demonstrated a strong depletion signal and functional relevance to DNA repair pathways, as synthetic lethality typically arises from the disruption of compensatory repair mechanisms. Interestingly, two out of the *MLH1* most promising depleted genes, *EXO1* and *PLK2*, have high connectivity with the MMR pathway as was demonstrated by utilising STRING analysis (https://string‐db.org/) (Figure 2C). *EXO1*, a known participant in the MMR pathway, and *PLK2*, involved in the cell cycle, were selected based on their strong functional association with DNA repair, making them prime candidates for SL interactions with *MLH1*. Moreover, they show high connectivity with the *WRN*, *PINK1*, and *POLB* genes (Figure 2C), which were previously suggested to have SL interaction with *MLH1* [33, 34, 35]. These genes also came up in our screen, and despite having moderate CS values (Figure 2A), *WRN* and *POLB* genes were two of the highly ranked SL hits among the DNA repair genes (Figure S2A). An additional high‐ranking gene in the *MLH1* candidate list was *NR5A2*, which had the highest statistical significance. *NR5A2*'s involvement in cellular metabolism and transcription regulation also suggests potential intersections with DNA damage response pathways, further supporting its candidacy for synthetic lethality in the *MLH1* model.

GSEA and STRING analysis on the *TP53* depleted gene list revealed pathways closely associated with *TP53*, including “signalling by Rho GTPases”, “cell cycle”, and “cellular senescence” (Figure 2B‐ii, and Figure S2B). “Signalling by Rho GTPases” emerged as the most significant pathway, featuring 18 genes significantly depleted in our screen (Figure 2B‐i), with *MYH10* standing out as the most noteworthy gene. Motivated by these results and to reduce the number of potential hits for validation, we proceeded to a secondary custom CRISPR‐Cas9 screen. The secondary custom‐made screen included the 132 genes identified in the genome‐wide screen along with additional 93 genes serving as positive and negative controls. *MYH10* displayed significantly greater depletion compared to other tested genes (Figure 2D).

In total, four genes were selected for further validation experiments *EXO1*, *NR5A2*, and *PLK2* for *MLH1* and *MYH10* for *TP53*. All of the genes demonstrated uniform trends among all sgRNAs and a clear separation from the WT screen results at the last time‐point (Figure 2E,F).

### 2.3 Genetic and Chemical Validation of SL Candidate Genes in Haploid hESC *MLH1* Deficient Model

**FIGURE 3:**
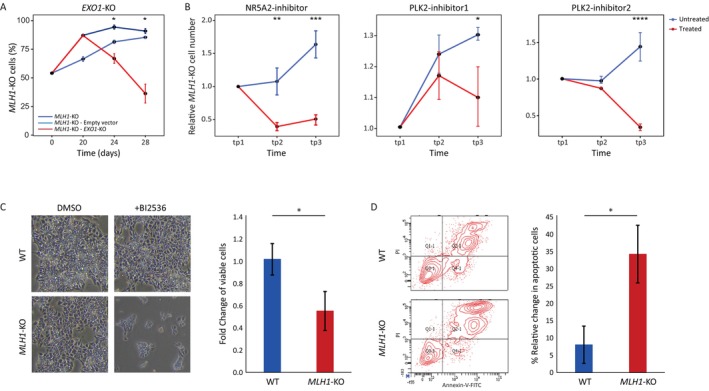
Genetic and chemical validation of synthetic lethality candidate genes in haploid hESCs *MLH1* deficient model. (A). Haploid *MLH1*‐KO hESCs were mixed with WT‐GFP cells in a 50:50 ratio. The mixed cells were infected with empty *CAS9* lentivector (blue), and *EXO1* sgRNA CAS9 lentivector (red). Control uninfected cells (dark blue). Changes in WT vs. *MLH1*‐KO dynamics were monitored at various time points using FACS. The percentages of the *MLH1*‐KO cells are represented. Dots represent the mean of biological replicates, and error bars represent the standard error of the mean (SEM). Comparison of the *MLH1*‐KO—Empty vector versus the *MLH1*‐KO—*EXO1*‐KO cells was performed by one‐sided *t*‐test, *p* = 0.03 for day 24 and *p* = 0.039 for day 28. (B). A mixture of WT‐GFP and *MLH1*‐KO cells, in a 50:50 ratio, was cultured both with (red lines) and without (blue lines) NR5A2‐inhibitor—SR1848 (left), PLK2‐inhibitor 1—ON1231320 (middle), and PLK2 inhibitor 2—BI2536 (right). The dynamic of *MLH1* mutant cell abundance in the culture was monitored over time using FACS and normalised to the initial time point. Without treatment, *MLH1*‐KO cells gradually dominate the culture, while their abundance significantly decreases under treatment. Dots represent the mean of replicates, and error bars represent SEM. The comparison was done by a one‐sided *t*‐test for each time point (tp): NR5A2‐inhibitor: *p* = 0.00371 and *p* = 0.00049 for SR1848 treatment in tp2 (Days 7–12) and tp3 (Days 17–22), respectively, *n* = 4 for both control and SR1848 treatment in either tp2 or tp3; PLK2‐inhibitor 1: *p* = 0.145 and *p* = 0.019 for ON1231320 treatment in tp2 (Days 2–7) and tp3 (Days 13–17), respectively, *n* = 4 for both control and ON1231320 treatment in either tp2 or tp3; PLK2‐inhibitor 2: *n* = 3 or 2 for control or BI2536 treatment in tp2 (Day 5), respectively, and *n* = 4 for both control and BI2536 treatment in tp3 (Day 10–15), *p* = 4.8E‐05 for BI2536 treatment in tp3. (C). Haploid hESCs with (*MLH1*‐KO) and without (WT) mutation in the *MLH1* gene were treated with BI2536. Images depicting treated and untreated (DMSO) cells are displayed on the left, with quantitative analysis of viable cell count provided on the right (*n* = 3, *p < 0.05, by two‐sided *t*‐test). (D). Annexin‐V‐PI staining, utilised for apoptosis detection, is depicted in the FACS density‐contour plot (left) of WT/*MLH1*‐KO cells treated with BI2536. Quantitative analysis of apoptotic cells provided on the right (*n* = 3, *p < 0.05, by two‐sided *t*‐test).

The analysis of the *MLH1* screen yielded three main hits (Figure 2E). Among them, *NR5A2* and *PLK2* are associated with small molecule inhibitors, whereas *EXO1* lacks such inhibitors, requiring different validation approaches. To demonstrate an SL interaction between the *EXO1* and *MLH1* genes, CRISPR‐Cas9 technology was employed to target *EXO1* in both WT and *MLH1*‐KO cells. Haploid WT‐GFP ESCs were mixed with haploid *MLH1*‐KO cells in a 1:1 ratio and subjected to three groups: (1) Untransduced cells, (2) Cells transduced with an empty Cas9 lentiviral vector, and (3) Cells transduced with Cas9 lentiviral vector containing *EXO1*‐sgRNAs. The infected populations of cells were grown in the presence of the selection antibiotic puromycin. Changes in the relative percentage of *MLH1*‐KO cells were monitored using fluorescence‐activated cell sorting (FACS) and are summarised in Figure 3A. In the control cultures (*MLH1*‐KO and *MLH1*‐KO—Empty vector), there was a gradual takeover of *MLH1*‐KO cells, constituting 85% and 90% of the population, respectively (Figure 3A). This observation aligned with a prior study indicating a selective advantage of *MLH1*‐KO pluripotent cells over WT cells [36]. Notably, targeting the *EXO1* gene (*MLH1*‐KO—*EXO1*‐KO) resulted in a significant decrease in the *MLH1*‐KO cell population, with a *p*‐value of 0.039, providing support for synthetic lethal interaction between the *EXO1* and *MLH1* genes (Figure 3A).

Utilising chemical inhibitors has a significant advantage, as the small molecules used, exhibit substantial penetrance into the cells and directly inhibit the gene product. In this context, three molecules that inhibit either NR5A2 or PLK2 were selected—SR1848 (MCE‐HY115613) an inhibitor of NR5A2, and ON1231320 (MCE‐HY100789) and BI2536 (MCE‐HY50698) two independent inhibitors of PLK2. Similar to the previously described genetic validation of *EXO1*, a cell competition assay of mixed culture containing haploid WT‐GFP ESCs and *MLH1*‐KO cells in a 1:1 ratio was used. Cells were cultured with or without chemical inhibitors and monitored utilising FACS. As predicted, untreated cells demonstrated a decline of WT‐GFP signal and a take‐over of the *MLH1*‐KO cells. In contrast, inhibiting NR5A2 or PLK2 with SR1848 or BI2536, respectively, resulted in a nearly complete take‐over of WT‐GFP cells (Figure 3B). The PLK2‐inhibitor ON1231320 exhibited a more modest, yet significant effect as well (Figure 3B).

To confirm the selective effect of the small molecules on *MLH1*‐KO hESCs, microscopy visualisation and quantification of both treated and untreated WT and *MLH1*‐KO cells were performed. The results revealed a notable sensitivity of the *MLH1*‐KO cells, with no visible effect observed on WT cells (Figure 3C and Figure S3A). An additional complementary assay assessing the apoptotic effect of the inhibitors on WT and *MLH1*‐KO haploid cells was undertaken. FACS‐based annexin assay presented a significant increase in apoptosis in the *MLH1*‐KO compared to WT cells (Figure 3D, Figure S3B). In summary, the validation in haploid hESCs with selected chemical inhibitors confirmed the role of both *NR5A2* and *PLK2* as promising *MLH1*‐SL targets.

### 2.4 Chemical Validation of SL Candidate Genes in *MLH1* Deficient Cancer Models

**FIGURE 4:**
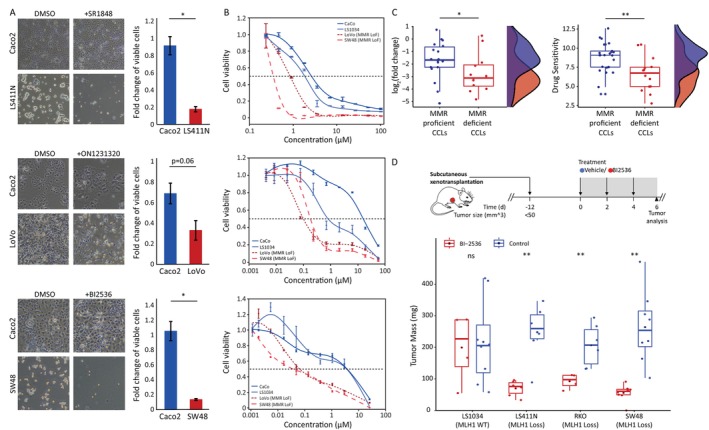
Chemical validation of synthetic lethality candidate genes in *MLH1* deficient cancer models. (A). CCLs with (LS411N, LoVo, SW48) and without (Caco2) MMR deficiency were treated with SR1848 (top) ON1231320 (middle), and BI2536 (bottom), for 4 days. Representative images of treated and untreated (DMSO) CCLs are displayed (left), with quantitative analysis (right) (*n* = 3, *p < 0.05). (B). Dose–response curves of DNA MMR deficient (red) and proficient (blue) CCLs treated with SR1848 (top) ON1231320 (middle) and BI2536 (bottom). Dots represent the mean of triplicates. Error bars represent the standard error of the mean (SEM) of triplicates. (C). Bioinformatic analysis of CCLs for treatment with BI2536—box plot and density plot of MMR‐proficient CCLs (blue), and MMR‐deficient CCLs (red). The left panel is the sensitivity data obtained from the Broad Repurposing Library and the PRISM multiplexed cell‐line viability assay. The y‐axis is log2 of the ratio of the abundance of cells in the treatment group vs. cells in the control DMSO group. The right panel is the sensitivity data obtained from the CTD2 database. Shown are box plot representations, with the median as the centre line, and 25% and 75% as box limits, *n* = 18 and 12 for MMR‐proficient CCLs and MMR‐deficient CCLs presented on the left panel, respectively; *n* = 25 and 14 for MMR‐proficient CCLs and MMR‐deficient CCLs presented on the right panel, respectively. *p < 0.05, **p < 0.01, by Kolmogorov–Smirnov test. (D). Upper panel: Schematic illustration of the experiment—13 NOD‐SCID Il2rg−/− mice were subcutaneously injected with LS1034 (MMR proficient) or with SW48, RKO and LS411N (MMR deficient) cells. 12 days after xenotransplantation, mice were subjected to a 6‐day treatment with BI2536 (red, *n* = 4 for LS1034 and RKO cell lines, *n* = 6 for LS411N and *n* = 8 for SW48), or vehicle (blue, *n* = 8 for LS1034, and SW48, and *n* = 6 for RKO and LS411N). Tumour mass was measured following the treatment and is illustrated in box plots with the median as centre line, and 25% and 75% as box limits, black dots represent the mean. *p* = 0.815 for LS1034 (ns), *p* = 0.0038 for LS411N, *p* = 0.0073 for RKO and *p* = 0.0012 for SW48, two‐sided *t*‐test. **p < 0.01, ns = non‐significant.

Following the specific response observed in *MLH1*‐KO hESCs treated with the chemical inhibitors, we extended our investigation to assess this response in CCLs. Given the high prevalence of loss‐of‐function (LoF) of *MLH1* in colorectal cancer, our focus turned to validating SL interactions in colorectal CCLs. Specifically, Caco2 and LS1034 were chosen as the MMR‐proficient cell lines, while LS411N, LoVo, and SW48 were selected as MMR‐deficient cell lines. We treated these cells in triplicates with SR1848, BI2536, or ON1231320 for 3–4 days, after which we counted the viable cells. The results revealed a notable disparity in the response of MMR‐deficient and proficient cells to all three molecules (Figure 4A).

For a better understanding of the effect of each inhibitor on the CCLs, we conducted a dose–response assay. Two MMR‐proficient colorectal CCLs—Caco2 and LS1034, and two MMR‐deficient colorectal CCLs, LoVo and SW48, were exposed to varying doses of each small molecule to understand its impact on each CCL. Notably, for all three inhibitors, the IC50 values of MMR‐proficient CCLs were consistently at least 2‐fold greater than those of MMR‐deficient CCLs. This difference became even more pronounced, reaching an order of magnitude larger under the treatment of BI2536 (Figure 4B). From these findings, we infer that MMR‐deficient cell lines exhibit greater sensitivity to *NR5A2* and *PLK2* inhibitors compared to the proficient ones.

Following the encouraging in vitro results, especially with BI2536 treatment, we aimed to extend the support for this small molecule by increasing the number of CCLs in our analysis. We obtained drug response data for BI2536 treatment on colorectal CCLs from the Broad Repurposing Library (https://repo‐hub.broadinstitute.org/repurposing) and the PRISM multiplexed cell‐line viability assay (https://www.theprismlab.org/) and from the CTD2 database. Colorectal CCLs were divided into two groups: MMR deficient (*n* = 12) and proficient (*n* = 18), and their sensitivity to BI2536 was compared. In both datasets, we noted a significant sensitivity of MMR‐deficient CCLs compared to the proficient ones (Figure 4C). When performing the same analysis using the two “gold standard” drugs typically utilised in colorectal cancer treatment, namely, fluorouracil (5‐FU) and oxaliplatin, no variations in the response between MMR‐deficient and proficient CCLs were found (Figure S4). This suggests that selectively inhibiting *NR5A2* or *PLK2* triggers cell death in MMR‐deficient cells, implying a potential SL with MMR deficiency.

Driven by our findings, we initiated an in vivo experiment to assess the impact of BI2536 on cancer growth in both MMR‐deficient (SW48, RKO & LS411N) and proficient (LS1034) cells. Xenotransplantation models in NOD‐SCID Il2rg−/− mice were established by subcutaneous injection of these colorectal CCLs. 12 days post‐injection, we started to treat with 50 mg/kg of BI2536 or vehicle (treatment control) every other day. After 6 days of treatment, tumour volumes were measured and analysed. The analysis revealed a significant inhibition of SW48, RKO and LS411N xenograft tumour growth by BI2536. In contrast, LS1034 xenografts did not display any noticeable differences in tumour growth (Figure 4D).

Following the validation of the genes identified in the genome‐wide CRISPR screen in hESCs, CCLs, and mice models, it becomes apparent that *EXO1*, *NR5A2*, and *PLK2* may demonstrate synthetic lethality with MMR deficiency.

### 2.5 Genetic and Chemical Validation of the SL Candidate *MYH10* Gene in a *TP53* Deficient Model

To confirm SL interactions between *MYH10* and *TP53*, a novel genetic validation assay that leverages the advantage of cell haploidy was developed. Thus, *TP53*‐KO and WT cells were transduced with a Cas9 lentiviral vector containing *MYH10*‐sgRNAs. Transfected cells were expanded, genomic DNA was isolated at different time points, and a library of the DNA sequences spanning the targeted *MYH10* locus was generated. The haploid nature of the cells allows a direct one‐to‐one correspondence of each sequence read with a specific cell in culture. This capability not only simplifies tracking mutation dynamics over time but, more importantly, aids in deducing their impact on cell viability. Thus, allowing the identification of SL interaction. This level of precision is out of reach in diploid or polyploid cells, where each cell contains a mix of mutations and WT sequences of the same locus, making it difficult to establish a clear connection between mutation dynamics and cell viability.

Sequencing results were analysed, and classified into frameshift (FS), Non‐Frame‐Shift (NFS), or WT. The frequency of these classifications for each sample, relative to the total sample count, was computed, enabling an analysis of trends between the WT and *TP53*‐KO groups. To ensure the robustness of the cut site locus, a quality control check was performed by examining mutation locations. 81% of all mutations clustered around the cut site locus, indicative of the efficiency of CRISPR‐Cas9 activity (Figure S5A).

**FIGURE 5:**
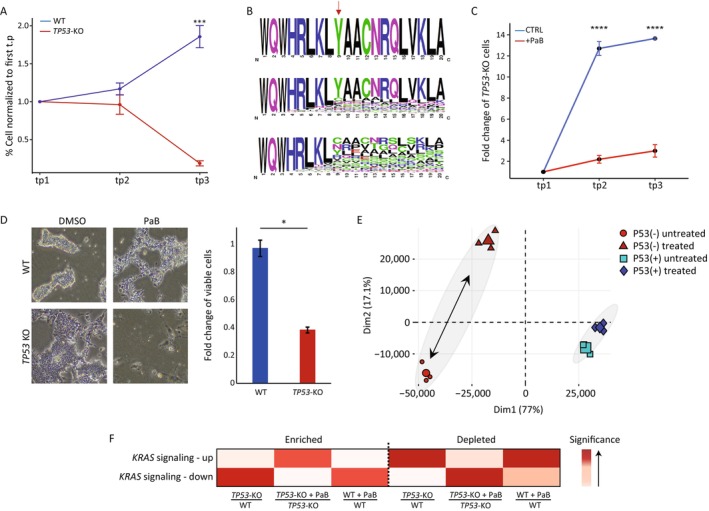
Genetic and chemical validation of the synthetic lethality candidate gene *MYH10*, in haploid hESCs *TP53* deficient model. (A). Comparison of LoF mutation distribution at different time points following the introduction of mutations to the *MYH10* gene in both haploid WT (blue) and *TP53*‐KO (red) cells. *n* = 138 different LoF mutations. The Y‐axis is the averaged normalised values according to the first time point (tp), that is, the first tp is equal to 1, and the other tps are the fold‐change that is relative to tp 1. ***p < 0.001. Dots represent the mean of biological replicates, and error bars represent SEM. (B). Visual depiction of the MYH10 amino acid sequence surrounding the CRISPR‐Cas9 cut site (red arrow). The original amino acid sequence is presented at the top (WT), while variations in the sequence for non‐frameshift mutations (*n* = 955) and frameshift mutations (*n* = 1551) are at the middle and the bottom, respectively. (C). A mixture of *TP53*‐KO and WT‐GFP cells, in a 1:99 ratio, was cultured both with (red lines) and without (blue lines) para‐amino‐blebbistatin treatment (+PaB). The progression of *TP53* mutant cell abundance in the culture was continuously monitored over time using FACS and normalised to the initial time point. Without treatment, *TP53*‐KO cells gradually dominate the culture, while their abundance significantly decreases with the application of treatment. The comparison was performed by a one‐sided *t*‐test for each time point (tp1‐3, representing day 0, days 9–15 and days 20–24). The *p*‐values for tp2 and tp3 are 3.0E‐08 and 3.9e‐05, respectively. **p < 0.01, ***p < 0.001. For tp2 and tp3 *n* = 6 for control and *n* = 4 for PaB treatment. (D). Haploid hESCs with (*TP53*‐KO) and without (WT) mutation in the *TP53* gene were treated with para‐amino‐blebbistatin for 4 days. Images depicting treated and untreated (DMSO) cells are displayed on the left, with quantitative analysis of viable cell count provided on the right (*n* = 3, *p < 0.05, two‐sided *t*‐test). (E). Principal Component Analysis (PCA) was performed on RNA‐seq results, comparing WT and *TP53*‐KO haploid hESCs treated or untreated with para‐amino‐blebbistatin for 4 days. (F). Heat map displaying False Discovery Rate (FDR) values of Gene Set Enrichment Analysis (GSEA), comparing wild‐type (WT) and *TP53*‐KO haploid hESCs treated or untreated with para‐amino‐blebbistatin for 4 days. Genes with increased expression were labelled as “enriched,” while those with decreased expression were labelled as “depleted.” The various comparison groups are specified beneath the heat map.

Our robust assay enabled the identification of 138 different LoF mutations, each found in both WT and *TP53*‐KO cells. Comparing their distribution at different time points revealed an increasing percentage of cells with LoF mutations over time in WT cells, whereas *TP53*‐KO cells displayed a decreasing frequency in LoF mutations (Figure 5A; *t*‐test; *p* < 1.6E‐21). The 5 most frequent LoF mutations showed a similar trend (Figure S5B). Collectively, these mutations represented ~90% of all LoF mutations, suggesting a preference for specific corrections at that cutting site location. As expected, amino acid sequence analysis of both FS and NFS mutations showed a moderate variation in NFS mutations and substantial variation in FS mutations starting from the cutting site area, further supporting the impact of LoF mutations on the *MYH10* gene (Figure 5B).

To further validate the synthetic lethal interaction between the *MYH10* and *TP53*, chemical inhibition validation was conducted. Utilising the small molecule para‐amino‐blebbistatin (PaB), a derivative of blebbistatin known to inhibit MYH10, a cell competition assay was implemented in conjunction with chemical inhibition as previously described. As expected, untreated cells showed a decline in the WT‐GFP signal, with *TP53*‐KO cells gradually taking over. In contrast, inhibition of MYH10 with PaB blocked the take‐over of *TP53*‐KO cells (Figure 5C).

To confirm the selective targeting of *TP53*‐KO hESC without affecting WT cells, microscopy visualisation and quantification of treated and untreated cells for both WT and *TP53*‐KO were conducted. The findings revealed a sensitivity of *TP53*‐KO cells, with no visible effects observed in WT cells (Figure 5D). To further demonstrate the differential response of *TP53*‐KO cells to MYH10 inhibition, we performed RNA‐seq on both WT and *TP53*‐KO hESCs after 4 days of treatment with PaB, alongside untreated controls. Principal component analysis (PCA) was employed to visualise the global transcriptional differences across these conditions. As shown in Figure 5E, the PCA plot reveals minimal separation between the treated and untreated WT cells, suggesting that PaB treatment alone does not substantially alter the transcriptomic profile of WT cells. In contrast, *TP53*‐KO cells exhibit a marked divergence between treated and untreated groups, indicating a significant transcriptional shift in response to PaB treatment (Figure 5E). This differential clustering supports our hypothesis that MYH10 inhibition has a more pronounced effect on *TP53*‐KO cells, aligning with the observed phenotypic differences. Gene Set Enrichment Analysis (GSEA) was performed, comparing several different groups: *TP53*‐KO vs. WT, which serves as a background to see the basic differences between WT and *TP53*‐KO cells; *TP53*‐KO treated with PaB vs. untreated *TP53*‐KO, which will show the effect of treatment with PaB on *TP53*‐KO cells; and WT treated with PaB vs. untreated WT, which will enable the identification of changes originating from treatment with PaB. GSEA with the 200 most enriched or depleted genes was employed. Among the pathways that were enriched/depleted in the *TP53*‐KO‐PaB vs. *TP53*‐KO comparison while showing the opposite trend or no trend in the other groups were the androgen response, several hormonal pathways, and TGF‐beta signalling (Figure S5C). Interestingly, the KRAS‐signalling pathway showed complementary results for genes that are enriched or depleted upon the activation of KRAS signalling—in the *TP53*‐KO‐PaB vs. *TP53*‐KO comparison, KRAS‐signalling up was significantly enriched, while in the WT‐PaB vs. WT and *TP53*‐KO vs. WT comparisons, KRAS‐signalling down showed significant enrichment. The examination of the most depleted genes showed the exact opposite trends (Figure 5F).

**FIGURE 6:**
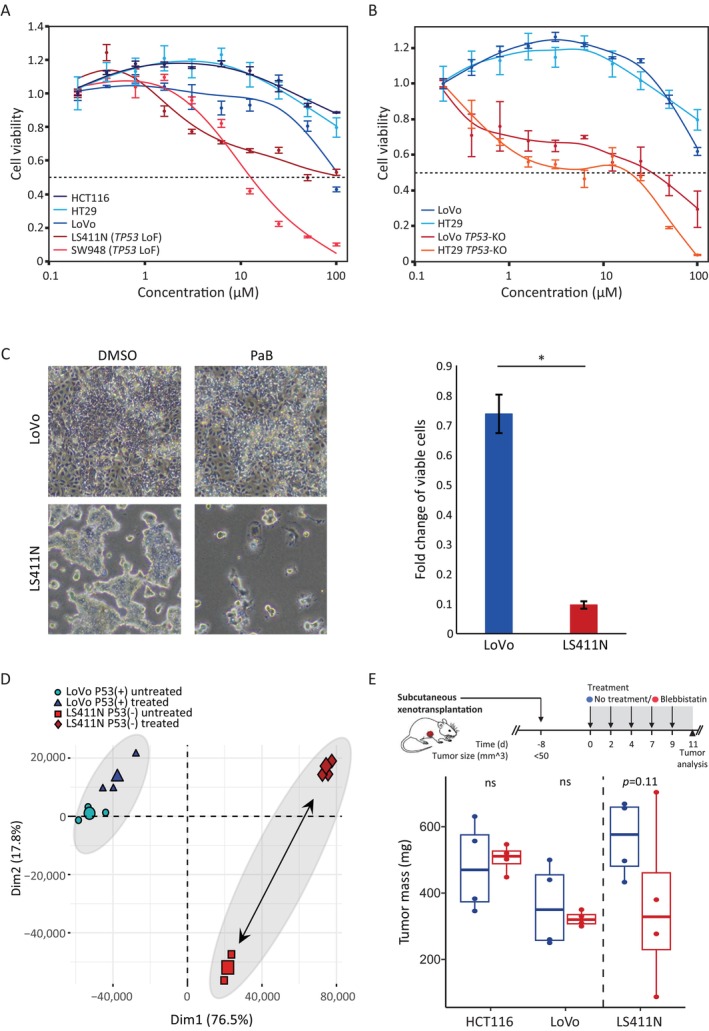
Genetic and chemical validation of the synthetic lethality candidate gene *MYH10*, in *TP53* deficient cancer models. (A). Dose–response curves of *TP53*‐LoF (red) and *TP53*‐WT (blue) CCLs treated with blebbistatin for 3–6 days. Dots represent the mean of triplicates. Error bars represent the SEM of triplicates. (B). LoVo and HT29 cell lines, originally without TP53‐LoF mutation, underwent *TP53* knockout using CRISPR‐Cas9 technology to generate isogenic cell lines. Dose–response curves of *TP53*‐KO (red) and *TP53*‐WT (blue) CCLs treated with blebbistatin for 3–6 days are depicted. The dots represent the mean of triplicates, with error bars indicating SEM for triplicates. (C). CCLs with (LS411N) and without (LoVo) *TP53*‐LoF were treated for 4 days with para‐amino‐blebbistatin. Left panel—representative images of treated and untreated (DMSO) CCLs. Right panel—cell count quantitative analysis (*n* = 3, *p < 0.05, two‐sided *t*‐test). (D). PCA was performed on RNA‐seq results to compare WT (LoVo) and *TP53*‐LoF (LS411N) CCLs treated or untreated with para‐amino‐blebbistatin for 4 days. (E). Left panel: Schematic illustration of the experiment—NOD‐SCID Il2rg−/− mice were subcutaneously injected with HCT116 (*TP53*‐WT, *n* = 8), LoVo (*TP53*‐WT, *n* = 8) and LS411N (*TP53*‐LoF, *n* = 8) CCLs. 8 days after xenotransplantation, mice were subjected to an 11‐day treatment with blebbistatin (red) or vehicle (blue). *N* = 4 for all different conditions. Tumour analysis at the endpoint was conducted, and their masses are illustrated in box plots with median as centre line, and 25% and 75% as box limits. *p* = 0.63 for HCT116, 0.29 for LoVo and 0.11 for LS411N, one‐sided *t*‐test.

Taken together, genetic and chemical validations in hESCs confirmed the SL interaction between *MYH10* and *TP53*. To demonstrate this SL interaction in cancer model, CCLs with and without *TP53* LoF mutations were used (Table S3). Blebbistatin dose–response assays demonstrated that CCLs with *TP53*‐LoF mutations (LS411N and SW48) were significantly more sensitive than CCLs without *TP53*‐LoF mutations (HCT116, HT29, and LoVo) (Figure 6A). To demonstrate the direct effect of blebbistatin treatment on *TP53* deficient cells, isogenic CCLs to LoVo, HT29, NCIH460, and Calu‐6 were established. All four CCLs are sensitive to Nutlin‐3 treatment (Figure S6A), thus facilitating the introduction of LoF mutations to the *TP53* gene utilising our *TP53* sgRNA vector and Nutlin‐3 selection. Blebbistatin treatment demonstrated the sensitivity of *TP53*‐KO cells versus their isogenic non‐LoF counterparts (Figure 6B, Figure S6B). Collectively, *TP53*‐deficient CCLs are significantly more sensitive to blebbistatin treatment (Figure S6B,C,D).

To confirm the selective effect of PaB treatment on *TP53* deficient cells, microscopy visualisation and quantification of both LoVo (*TP53* non‐LoF) and LS411N (*TP53*‐LoF) were performed following a three‐day treatment. The results revealed a notable sensitivity of LS411N cells, with no visible effect observed on LoVo cells (Figure 6C).

To support the differential effect of *MYH10* inhibition on *TP53*‐deficient CCLs, RNA‐seq of WT‐*TP53* (LoVo) and *TP53*‐deficient (LS411N) CCLs with or without treatment with PaB for 4 days was performed. PCA of RNA‐seq results indicated almost no difference between the treated and untreated LoVo cells, while a substantial difference was observed between the treated and untreated LS411N cells (Figure 6D), indicating a drastic transcriptomic change solely in PaB‐treated *TP53*‐deficient cells, and thus validating our results in haploid hESCs.

The supportive in vitro results in both hESCs and CCLs were followed by an in vivo experiment to assess the impact of blebbistatin on cancer growth in both *TP53*‐deficient (LS411N) and proficient (HCT116 and LoVo) cells. Xenotransplantation models in NOD‐SCID Il2rg−/− mice were established by subcutaneous injection of these CCLs. 8 days post‐injection, we started to treat with 50 mg/kg of blebbistatin or vehicle (treatment control) every other day. After 11 days of treatment, tumour volumes were measured and analysed. The analysis revealed inhibition of LS411N xenograft tumour growth by blebbistatin, with a borderline significant *p*‐value of 0.1. In contrast, both HCT116 and HT29 xenografts did not display any noticeable differences in tumour growth, with non‐significant *p*‐values (Figure 6E). In summary, our diverse and complementary validations demonstrate an SL interaction between *MYH10* and *TP53* genes.

## 3 Discussion

This paper presents an innovative approach for identifying synthetic lethal interactions by leveraging genome‐wide genetic screening in haploid hESCs. Through the application of this method, novel synthetic lethal interactions linked to LoF mutations in the *MLH1* and *TP53* genes were successfully identified. To underscore the robustness of this innovative approach, the newly discovered targets underwent rigorous validation in diverse cancer models, demonstrating the efficacy of the methodology.

hESCs were previously used to model different diseases and cancer in particular [37]. They were used to model tumorigenicity aspects of retinoblastoma [38], Li‐Fraumeni syndrome [39], and MMR deficiency [36, 40]. Building on these precedents, models for both MMR and p53 deficiencies in haploid hESCs were established and employed in genome‐wide genetic screenings to uncover novel synthetic lethal interactions. As previously mentioned, CCLs are commonly used for this purpose but have intrinsic disadvantages that can be effectively addressed by the application of haploid hESCs. Notably, polyploidy emerges as a major drawback in CCLs, stemming from their intrinsic genomic instability. The identification of a potential SL interaction hit in CCLs requires complete CRISPR‐Cas9‐mediated LoF mutations in all gene copies. However, the probability of such an event decreases significantly with increased gene copy number, leading to false negative results. Conversely, polyploidy in CCLs can induce multiple double‐strand breaks, potentially causing cell cycle arrest and apoptosis, ultimately resulting in the identification of false positive targets [41, 42]. Collectively, these factors contribute to both false negative and positive results, weakening the reliability of genetic screening outcomes in CCLs. Furthermore, the presence of multiple point mutations and genomic aberrations in CCLs adds complexity to the genetic landscape, making the consistent reproduction of results across different cell lines challenging [42, 43]. These drawbacks emphasise the superiority of haploid hESCs that have only one copy and a “clean” genetic background [13].

The focus of this study was directed to mutations resulting in the loss of MLH1 and p53 proteins, frequently observed in colorectal cancer (CRC) and ranks as the second most prevalent contributor to cancer‐related deaths. Thus, facilitating subsequent validation endeavours within CRC models. Previously, three genes—*WRN*, *POLB*, and *PINK1*—were suggested to have a synthetic lethal (SL) interaction with the *MLH1* gene. Among them, the WRN helicase was identified through successful genome‐wide genetic screenings in CCLs [33] and confirmed using different cancer models in subsequent studies [44]. These findings enable testing the capacity of genetic screening in haploid hESCs as a novel approach for uncovering SL interactions to specifically target tumour cells. Thus, all the previously identified genes came up in our *MLH1* screen. The *WRN* and *POLB* genes were recognised as two of the highly ranked SL hits among the DNA repair genes (Figure S2). In addition to known hits, the screen uncovered three novel hits that were top rank in this study, *PLK2*, *NR5A2*, and *EXO1*. These novel hits were successfully validated both in hESCs and CRC models (Figures 3 and 4). Two of the novel hits, *PLK2* and *EXO1*, demonstrated high connectivity with the WRN helicase, *POLB*, and the MMR pathway genes (Figure 2), suggesting a similar SL mechanism of action. Utilising chemical inhibitors for PLK2 and NR5A2, the SL interaction with MLH1 was further demonstrated in vitro and in vivo utilising CCLs (Figures 3 and 4).

Genome‐wide genetic screening in CCLs for SL interaction with *TP53* presents a challenge since many of them carry a mutation in *TP53* or other genes in its pathway and therefore don't have a proper WT *TP53* control. To address this challenge a novel model for p53 deficiency was established in haploid hESCs and used in a genome‐wide genetic screening (Figure 1). To further explore the derived data and pinpoint the best candidate gene, a follow‐up targeted screen was performed on the top candidate and control genes. Thus, we could identify the *MYH10* gene as a novel SL hit for the *TP53* gene (Figure 2). A rigorous genetic validation was performed in haploid hESCs demonstrating the SL interaction of *MYH10* and *TP53* in hESCs (Figure 5). The availability of small molecule inhibitors to *MYH10*, blebbistatin and its derivative para‐amino blebbistatin, enabled following up with chemical validation assays both in hESCs and in CCLs (Figures 5 and 6). These chemical validation assays demonstrated differential molecular response due to *TP53* LoF mutation, high sensitivity in dose–response curves, viability, and in vivo xenograft assays (Figures 5 and 6). The dose–response curves demonstrated an effect only in high‐range concentrations suggesting that the development of a novel and more effective small molecule is needed to improve animal study results and test a possible clinical use.

RNA‐seq analysis suggested that KRAS signalling is decreased upon MYH10 inhibition or LoF mutations in the *TP53* gene. However, when MYH10 is inhibited in p53 deficient cells the KRAS signalling is activated (Figure 5). KRAS, a well‐known oncogene within the RAS protein family, plays a crucial role in autophagy and apoptosis [45]. Given this intriguing pattern of KRAS signalling and its pivotal functions in cellular processes, investigating the causal connection of KRAS signalling, p53 deficiency, and MYH10 inhibition could be of interest and might help uncover the SL mechanism of action.

A possible mechanism of action to the *TP53*‐*MYH10* SL interaction might be related to the central role that these genes have in the progression of the cell cycle. p53 regulates cell cycle progression and is involved in multiple checkpoints including cell cycle arrest due to disruption in cytokinesis [46]. *MYH10* and its paralog *MYH9*, have a crucial role in cytokinesis. These paralogs, which have partially redundant biological properties, interact during cytokinesis and are essential for the contraction of the actomyosin ring. Interestingly, there is a significant reduction in the expression of *MYH9* in *TP53*‐KO cells (Figure S6E). Thus, p53 deficient cells that have a reduced *MYH9* expression, might not be able to compensate for MYH10 inhibition and have a disruption in cytokinesis. In the absence of p53, there is no cell cycle arrest, and the disruption in cytokinesis cannot be resolved leading to cell death.

This research contributes to the expanding landscape of synthetic lethality studies and highlights the potential clinical relevance of haploid hESCs genetic screening in the context of cancer therapeutics. One of the most intriguing results with a potential imminent clinical application is the identification of the *PLK2* gene as the SL partner of *MLH1*. The small molecule BI2356, which reached clinical trials, was originally designed to inhibit PLK1 but also has an inhibitory effect on PLK2 [47]. In our research, the sensitivity of MMR‐deficient CCLs to BI2356 was demonstrated on a subset of lines (Figure 4). Moreover, the sensitivity of MMR‐deficient CCLs to BI2356 was demonstrated utilising open data from the Broad and NIH institutes. Thus, emphasising its potential for treating MMR‐deficient tumours.

## 4 Materials and Methods

### 4.1 CRISPR/Cas9 Mediated Genetic Manipulation

For model development‐ Cells were transiently transfected utilising X‐tremeGENE 9 DNA Transfection Reagent with pSpCas9(BB)‐2A‐GFP vector (PX458) vector (a gift from Feng Zhang, Addgene cat. no. 48138) with sgRNAs directed against *TP53* and *MLH1* (Table S2) according to manufacturer instructions. GFP‐positive cells were sorted and plated in serial dilutions to allow clonal selection. Genomic DNA was extracted from isolated clones, relevant loci were amplified via PCR, and sent for Sanger sequencing to validate the introduction of LoF mutations.

For candidate gene validating‐ *EXO1* and *MYH10* sgRNAs were selected based on the sgRNA's profile from our screens. All sgRNAs were cloned into the lentiCRISPR v2 lentiviral vector (a gift from Feng Zhang, Addgene cat. no. 52961). To produce the lentiviruses, 293 T cells were transfected with sgRNA‐containing lentiCRISPR v2, pCMV‐VSV‐G (a gift from Robert Weinberg, Addgene cat. no. 8454) and psPAX2 (a gift from Didier Trono, Addgene cat. no. 12260) plasmids at a ratio of 2:1:1.5 (10 mg total per plate), respectively, in the presence of polyethyleneimine “Max” (PEI‐Max) (Polysciences) at a 1:2 ratio of DNA to PEI‐Max. Transfection medium was exchanged with standard hESC growth medium (described above) after 16–24 h, and lentiviral particle‐containing culture supernatant was harvested 60–65 h after transfection. The culture supernatant was spun down at 3000 rpm for 10 min at 4°C and then filtered through 0.45 mm cellulose acetate filters (Millipore). The filtered supernatant was frozen in aliquots at −70°C. Haploid‐enriched hESC cultures were trypsinized with Trypsin–EDTA, centrifuged, and resuspended in hESC growth medium supplemented with 10 μM ROCK inhibitor Y‐27632 and 8 mg/mL polybrene (Sigma). The thawed viruses were then added to the cell suspension. Transduced cells were plated on feeder layer mouse embryo fibroblasts. 24 h after transduction, the virus‐containing medium was replaced with a standard hESC growth medium. 36–48 h after transduction, the medium of the cells was replaced with a medium that contained puromycin (0.3 mg/mL, Sigma). Cells were kept under antibiotic selection for 7–10 days.

In *TP53* mutant cells a GFP cassette was inserted into the end region of the tubulin gene using the CRISPR–HOT method (Artegiani et al. 2020).

Sequences for *EXO1*, *MYH10*, *MLH1*, and *TP53* are summarised in Table S2.

### 4.2 Western Blot

Cell extracts were prepared with cell lysis buffer (1% Triton X‐100, 150 mM NaCl, 50 mM Tris HCl, pH 7.4, 1 mM EDTA, and protein inhibitor cocktail (Sigma Aldrich Co. Rehovot, Israel)) on ice. Following SDS polyacrylamide gel electrophoresis (SDS‐PAGE) separation, proteins were transferred to nitrocellulose membranes and blocked with 5% low‐fat milk. Membranes were incubated with rabbit anti‐MLH1 (ab108622, Abcam), mouse anti‐p53 (#2524, Cell Signalling), rabbit anti‐GAPDH (ab9485, Abcam), or rabbit anti‐Tubulin (#2125, Cell Signalling), primary antibodies, washed with PBS containing 0.001% Tween‐20 (PBST) and incubated with the appropriate secondary antibody, mouse anti‐rabbit‐HRP, or goat anti‐mouse‐HRP. After washing in PBST, membranes were subjected to enhanced chemiluminescence detection analysis.

### 4.3 Mutational Signature Analysis

*MLH1*‐KO haploid hESCs and their WT counterparts were grown for three months to allow mutation accumulation, subclones of *MLH1*‐KO (*n* = 8) and WT (*n* = 3) hESCs were established and their genomic DNA was extracted with gSYNC DNA extraction kit (GeneAid). Libraries for Whole Genome Sequencing were constructed with a “PCR free” kit for WGS, Illumina. WGS was performed with NOVA‐Seq 6000, Illumina.

Whole‐genome FASTQ files were analysed using the Illumina “Dragen Somatic” pipeline to create Variant Call Format (VCF) files. VCF files were then converted to Mutation Annotation Format (MAF) files to obtain more information about each mutation. Next, the Cosmic sigProfiler was used for mutational signature analysis.

### 4.4 Synthetic Lethal (SL) Interaction Utilising CRISPR‐Cas9 Screens

300 million WT/*MLH1‐*KO/*TP53*‐KO haploid hESCs were trypsinized, centrifuged, and resuspended in mTeSR medium supplemented with 10 μM ROCK inhibitor (Y‐27632) and 8 μg/mL polybrene (Sigma). The Brunello ready‐to‐use Lentiviral pooled library (Addgene‐ # 73179‐LV) was calibrated to achieve 30%–50% infection efficiency that corresponds to a ~ 0.5–1 multiplicity of infection (MOI). The transduced hESCs were densely plated on Matrigel‐coated plates overnight (3 million cells in 1.5 mL hESC medium per well in a six‐well plate). 24 h after transduction, cells were passaged at a ratio of 1:6 in the presence of 5 μM ROCK inhibitor (Y‐27632). During this passaging, 50 million cells were harvested for DNA extraction and sgRNA analysis for the initial time point after infection. 48 h after transduction, cells were subjected to puromycin selection (0.3 mg/mL, Sigma) for 10 days before being passaged again. The cells were expanded for 21, 23, or 27 days (for WT, *TP53*, or *MLH1*, respectively), and samples containing 50 million cells were collected at five different time points, achieving a coverage of approximately 650x. Genomic DNA was isolated, and NGS libraries were constructed using unique primers identifying the pooled sgRNAs. The libraries were sequenced on the MiSeq platform from Illumina, generating ~60 million reads per sample.

### 4.5 SL Interaction Screens Analysis

Fastq files of the 3 different conditions were analysed using 3 different bioinformatic pipelines to identify depleted genes. The pipelines use different normalisation methods and significance tests. The output of each pipeline is (1) CRISPR Score (CS) which is calculated by the Avg(log_2_(cond. 1/ cond. 2)) for all different sgRNAs of a gene and (2) *p*‐value which is based on the sgRNAs distribution. By the end of this process, each gene has a CS and *p*‐value for 3 comparisons: (1) WT_day21 vs. WT_day1 which we'll refer to as “WT” (2) MLH1_day27/TP53_day23 vs. WT_day1 which we'll refer to as “MLH1” or “TP53” respectively (3) MLH1_day27/TP53_day23 vs. WT_day21 which we'll refer to as “MLH1*” or “TP53*” respectively. To obtain the most promising genes, which are depleted in the MLH1 and TP53 screens and enriched or do not significantly change in the WT screen, we applied several selection criteria. First, we removed genes that are not expressed in hESCs. The second selection criterion is based on the CS and *p*‐value of each gene. We defined the thresholds for depleted, enriched, and static conditions: depleted genes = genes with CS < −0.5 and *p*‐value < 0.05; enriched genes = genes with CS > 0.5 and *p*‐value < 0.05 and static genes = genes with CS between −0.5 and 0.5 and with *p*‐value < 0.05. We selected genes that show one of the following trends: depleted in MLH1*/TP53* and (1) static in the MLH1/TP53 and enriched in the WT or (2) depleted in MLH1 and enriched in the WT or (3) depleted in the MLH1/TP53 and static in the WT. Genes that passed the above thresholds in at least 2 of the pipelines were further analysed. In the final step, we kept genes in which the third quartile of the MLH1_day27/TP53_day23 sgRNAs is lower than the first quartile of WT_day1 and WT_day21 sgRNAs.

### 4.6 Focused Customised CRISPR Library

The virus amount was calibrated to achieve ~0.5–1 MOI. 60 million WT/*TP53‐*KO cells were infected with the LentiCrispr pooled customised library that was purchased from Vector Builder Ltd. and was designed to target 132 genes and 93 positive and negative control genes. Each gene was targeted by 8 different sgRNAs—a total of 1800 sgRNAs. 200 additional sgRNAs that target non‐coding loci complete the list to a total of 2000 sgRNAs (sgRNA sequences and gene list are presented in Table S1). One day post‐infection puromycin treatment was initiated to eliminate cells that were not properly infected with the virus. The cells were expanded for 24 days and samples containing 7 million cells were collected at 5 different time points—Day 1, Day 9, Day 14, Day 18, and Day 24. This allowed a x3,500 coverage. Genomic DNA was isolated and used to create a library for NGS. The libraries were sequenced on MiSeq, Illumina, with ~6×10^6^ reads per sample.

### 4.7 Annexin V/PI Analysis for Apoptosis Detection by Flow Cytometry

WT or *MLH1*‐KO haploid hESCs were treated with 10 nM BI2536 or 1.5uM SR1848. 3 days later, cells were harvested, washed with PBS, and resuspended in the binding buffer provided in the Annexin V‐FITC Apoptosis Detection Kit (MEBCYTO Apoptosis Kit, MBL). 5 μL of Annexin V‐FITC was added to 100 μL of cell suspension, and the mixture was incubated for 15 min in the dark at room temperature. After incubation, 400 μL of binding buffer and 5 μL of PI were added, and samples were analysed using a flow cytometer (ARIA). Unstained cells were used for fluorescence compensation. Quadrant gates were set based on unstained control to distinguish viable (Annexin V‐ and PI‐negative), early apoptotic (Annexin V‐positive and PI‐negative), and late apoptotic (Annexin V‐ and PI‐positive) cell populations.

### 4.8 Cell Viability Assessment

A total of 300,000 cells were plated in 6‐well plates and allowed to adhere for 4 h. Subsequently, the cells were treated with specific compounds: 10 nM BI2536, 500 nM ON123132, 1.5uM SR1848, or 15uM PaB, for 3–4 days. Following the treatment period, representative images of the cells were captured to document morphological changes. To assess cell viability, the cells were then collected, stained with trypan blue, and counted using a cell counter (Invitrogen).

### 4.9 Cell Competition Assay

To assess the effect of *EXO1* knock‐out on haploid hESC *MLH1*‐KO or WT cells, we applied the cell competition assay. In this assay, GFP‐labelled, *MLH1*‐KO cells were mixed in predetermined proportions with WT cells (1:1 ratio), and changes in the proportions of the labelled cells after viral transfection with lentiCRISPR v2 lentiviral vector (empty vector or with sgRNA against *EXO1*) were measured by FACS analysis.

### 4.10 Validation in CCLs

To further confirm candidate gene selections, CCLs were used to test the effect of the gene's chemical inhibitors. The complete list of CCLs used in this study appears in Table S3. All CCLs were purchased from the ATCC reservoir (https://www.atcc.org). All growing media and cell serum were purchased from the ATCC. CCL tissue culture maintenance and experimentation were performed following the protocols supplied by the vendor.

CCLs were selected based on their *TP53* and *MLH1* status, i.e. WT or LoF. For dose–response curve experiments, 5000–7000 cells per well (96‐well plates) were seeded in triplicates. Control and nine concentrations of specific chemical inhibitors (SR1848, BI2536, ON1231320, and Blebbistatin) were selected for dose–response assay. The effect of the inhibitors on the cell viability of the different CCLs was tested for 3–6 days. Cell viability was measured by using CellTiter‐Glo Luminescent Cell Viability Assay (# G7572, Promega). Inhibitor concentrations are summarised in Table S4.

### 4.11 Flow Cytometry

Cell samples for the analysis of GFP expression were spun down at 1500 g for 4 min and the cell pellet was gently resuspended in 2 mL FACS solution (phosphate‐buffered saline, 2% FCS). Live cells were discriminated from cell debris and dead cells based on physical parameters (forward‐ and side‐light scatter). Fluorescence background levels were set with GFP‐negative cells. Following harvesting, cells were filtered through a 70 μm cell strainer and analysed by flow cytometry (BD Biosciences FACSAria III) and Flowjo software (FlowJo LLC).

### 4.12 In Vivo Validations

All experimental procedures were approved by the ethics committee of the Hebrew University of Jerusalem. Selected CCLs (5 × 10^6^) were resuspended in 100 μL medium and 100 μL Matrigel and subcutaneously injected into NOD‐SCID Il2rg−/− immunodeficient mice. Tumours were excised 3 weeks after injection.

### 4.13 Bioinformatic Validation in Cancer Cell Lines

Data on CCLs sensitivity to BI2536 was downloaded from 2 sources: (1) The PRISM Repurposing which contains small molecule viability datasets generated using the Broad Repurposing Library and the PRISM multiplexed cell‐line viability assay [48] and (2) The Cancer Target Discovery and Development (CTD^2^). NetworkMMR‐deficient CCLs were defined as CCLs with Microsatellite instability (MSI) and with a mutation in one of the MMR genes, whereas MMR‐proficient CCLs were defined as CCLS with Microsatellite Stability (MSS) and without a mutation in any of the MMR genes.

### 4.14 NGS‐Based Validation in Haploid hESCs

The genetic assay was conducted in haploid hESCs by employing CRISPR‐Cas9 technology on target potential genes in both *TP53*‐KO and WT cells. The *MYH10* locus was sequenced in *TP53*‐KO and WT cells at 3 different time points across two unbiased experiments, each conducted in duplications. NGS results were analysed using SIQ, a tool that aligns and classifies the data to mutation types (deletion, insertion, mismatch, etc.) and provides additional details on each mutation, such as its specific location and length. Following this, we categorised each mutation into Frame‐Shift (FS), Non‐Frame‐Shift (NFS), or WT. The frequency of these classifications for each sample, relative to the total sample count, was computed, enabling an analysis of trends between the WT and *TP53*‐KO groups. To ensure the robustness of the cut site locus, a quality control check was performed by examining mutation locations. These locations were referenced to the PAM sequence, with zero denoting the position of the CRISPR‐Cas9 cut site. For indels longer than 1, the location was defined as the one nearest to the cut site locus.

Comparison of the distribution of *MYH10* LoF mutations at different time points in both WT and *TP53*‐KO cells was performed by observing the LoF mutations present in t.p1 in both WT and *TP53*‐KO (*n* = 138). To evaluate changes over time, each mutation was normalised to the initial t.p, making each t.p a fold‐change relative to t.p1. The most frequent LoF mutations were selected based on cumulative frequency across all samples. Weblogo was used to visualise all FS and NFS mutations.

### 4.15 RNA‐Seq Isolation and Analysis

Treated (15uM PaB for 4 days) and untreated control cells were collected and washed x2 with PBS. The cell pellet was used for RNA isolation with a Qiagen RNeasy kit.

Libraries for RNA‐seq were constructed and sequenced with MiSeq, Illumina.

Reads were aligned to the hg38 reference genome using the Rsubread r package and read counts were calculated using the featureCounts function in R. Quality control was performed using FASTQC. PCA was done using the prcomp function from the stat R package.

### 4.16 Gene Set Enrichment Analysis (GSEA)

GSEA was performed by comparing different groups and extracting the unique and with biological relevance, using the clusterProfiler package in R. Genes were ranked according to the fold‐change and *p*‐value of the different comparisons. The top 200 genes that were either depleted or enriched were used to find enriched pathways.

### 4.17 Statistical Analysis

Statistical analysis was performed using R statistical environment and Microsoft Office Excel. Comparisons of means were done using a *t*‐test. Comparison between the distribution of sensitivity data was tested using the Kolmogorov–Smirnov Test.

### 4.18 CTD2 Acknowledgement

The results shown here are, in part, based upon data generated by the Cancer Target Discovery and Development (CTD^2^) Network (https://www.cancer.gov/ccg/research/functional‐genomics/ctd2) established by the National Cancer Institute's Center for Cancer Genomics.

## Author Contributions

Rivki Cashman, Elyad Lezmi, Hagit Philip, Jonathan Nissenbaum, Oded Kopper, and Nissim Benvenisty designed the experiments; Rivki Cashman, Guy Haim‐Abadi, Elyad Lezmi, Hagit Philip, and Jonathan Nissenbaum performed the experiments with assistance from Ruth Viner‐Breuer, Chen Kozulin, Tamar Golan‐Lev, Aseel Gadban, Shiri Spinner‐Potesky, and Ofra Yanuka; Rivki Cashman, Elyad Lezmi, Hagit Philip, Jonathan Nissenbaum, Guy Haim‐Abadi and Oded Kopper wrote the manuscript with input from Nissim Benvenisty; Oded Kopper and Nissim Benvenisty supervised the work.

## Ethics Statement

All experiments were performed according to the ethical guidelines of the Hebrew University.

## Conflicts of Interest

Oded Kopper and Elyad Lezmi are V.P. R&D and Nissim Benvenisty is CSO of NewStem Ltd.

## Supporting information


Figures S1‐S6.

**Table S1.** List of all genes and their sgRNAs used for the custom CRISPR library.

**Table S2.** sgRNA sequences used for the generation of KO cell lines.

**Table S3.** List of CCLs and their TP53 status and MMR status used for validation.

**Table S4.** List of Inhibitors concentrations.

## Data Availability

The data that supports the findings of this study are available in the Supporting Information of this article.
