# Supplementary material for: Genome‐Wide Screening in Haploid Stem Cells Reveals Synthetic Lethality Targeting MLH1 and TP53 Deficient Tumours
Source: Cell Prolif. 2025 Jan 15;58(11):e13788. doi: 10.1111/cpr.13788 (PMC12584864; doi:10.1111/cpr.13788)
Supplement: Supplementary file 1 — Figures S1‐S6. [file CPR-58-e13788-s003.pdf]

Supplementary information

Genome-wide Screening in Haploid Stem Cells Reveals Synthetic Lethality Targeting MLH1 and TP53 deficient tumors

Rivki Cashman[1,#], Guy Haim-Abadi[2,#], Elyad Lezmi[1,2,#,] Hagit Philip[1,#], Jonathan Nissenbaum[1,#], Ruth Viner-Breuer[1], Chen Kozulin[1], Tamar Golan-Lev[2], Aseel Gadban[2], Shiri Spinner-Potesky[1], Ofra Yanuka[2], Oded Kopper[1],* and Nissim Benvenisty[1,2,*]

[1]NewStem LTD, P.O. Box 39173, Jerusalem 913910, Israel

[2]The Azrieli Center for Stem Cells and Genetic Research, Department of Genetics, Silberman Institute of Life Sciences, The Hebrew University, Jerusalem 91904, Israel.

[#]These authors contributed equally

[*]Co-correspondence

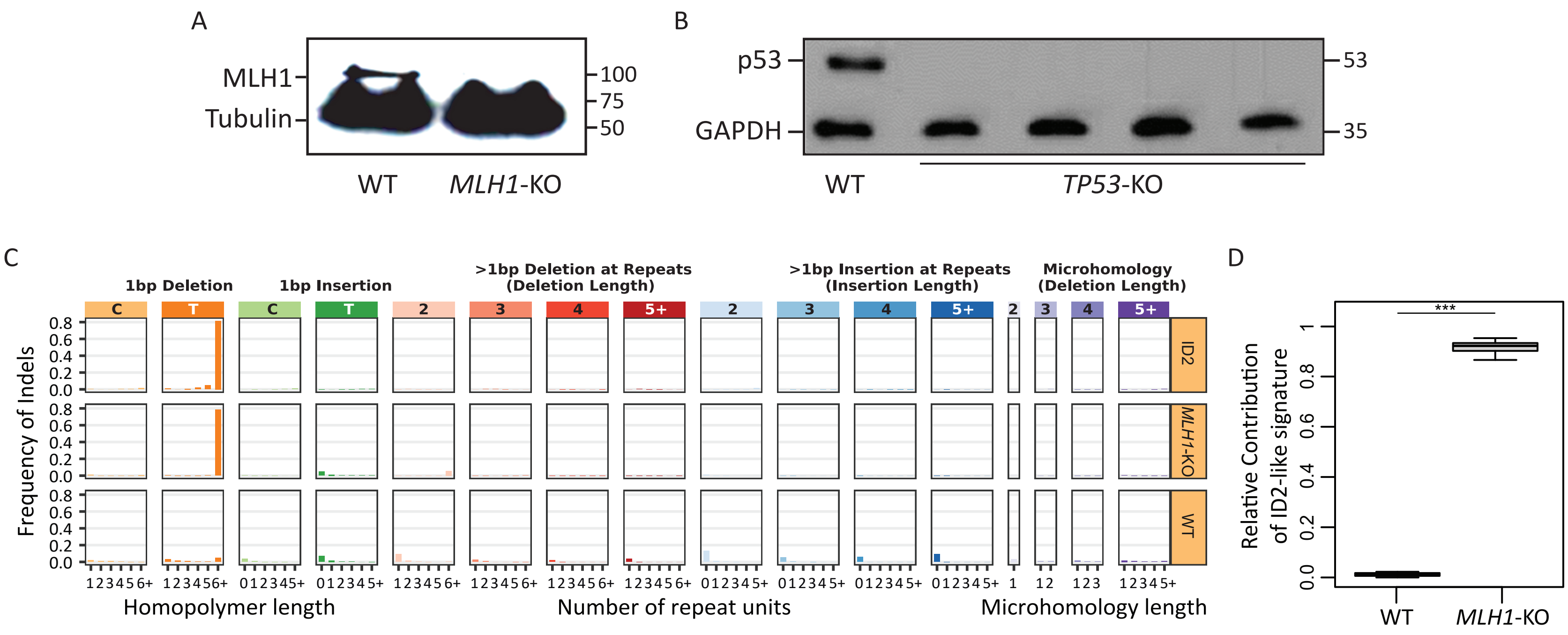

Supplementary Figure 1: Establishing and validating haploid models for synthetic lethality genome-wide screening.

A. Western blot analysis demonstrates loss of MLH1 protein.

B. Western blot analysis demonstrates loss of p53 protein in several clones (*TP53*-KO).

C. The similarity between the Indel 2 (ID2) signature (upper panel) to the *MLH1*-KO (middle panel) and WT cells (lower panel). The colors describe the length of the insertion/deletion, and the x-axis is a subclassification that describes the repeats of each insertion/deletion. The y-axis describes the frequency of each change.

D. Relative contribution of ID2-like signature to WT or *MLH1*-KO cells. Shown are box plot representations, with median as center line, and 25% and 75% as box limits, n=3 and 8 for wild type and *MLH1*-KO clones that were analyzed utilizing whole-genome sequencing, respectively. *** p<0.001, by two-sided T-test.

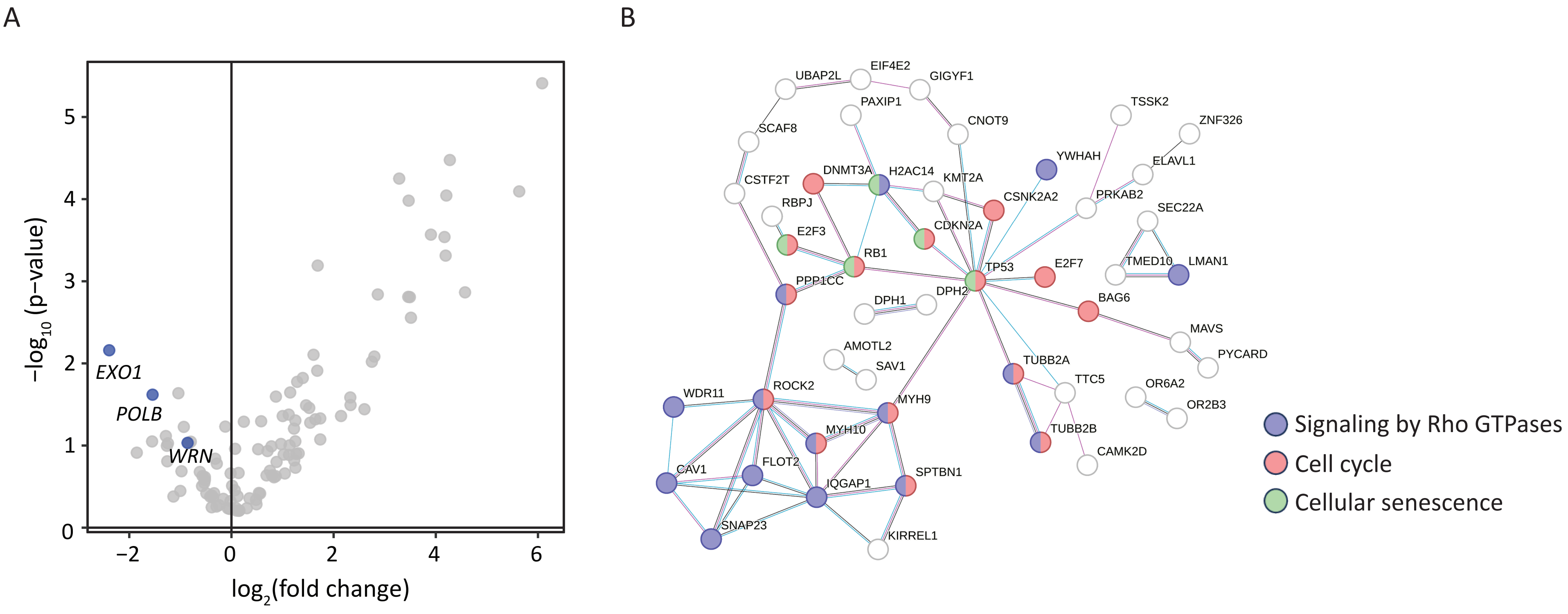

Supplementary Figure 2: Genome-wide and custom-made genetic screening for synthetic lethality interaction identification.

A. Volcano plot displaying the CRISPR score (CS = $\log_2$(fold change) of *MLH1*-KO last time point (tp) vs. WT last tp) and adjusted p-values for DNA repair genes in the genome-wide *MLH1*-KO screen. Blue dots = *EXO1*, *POLB*, and *WRN*.

B. The most significant genes in the genome-wide *TP53*-KO screen, were analyzed for protein interactions STRING resource. Genes' nodes color in STRING. The colored nodes categorize genes based on the pathways analyzed in gene set enrichment analysis (Figure 2D-ii).

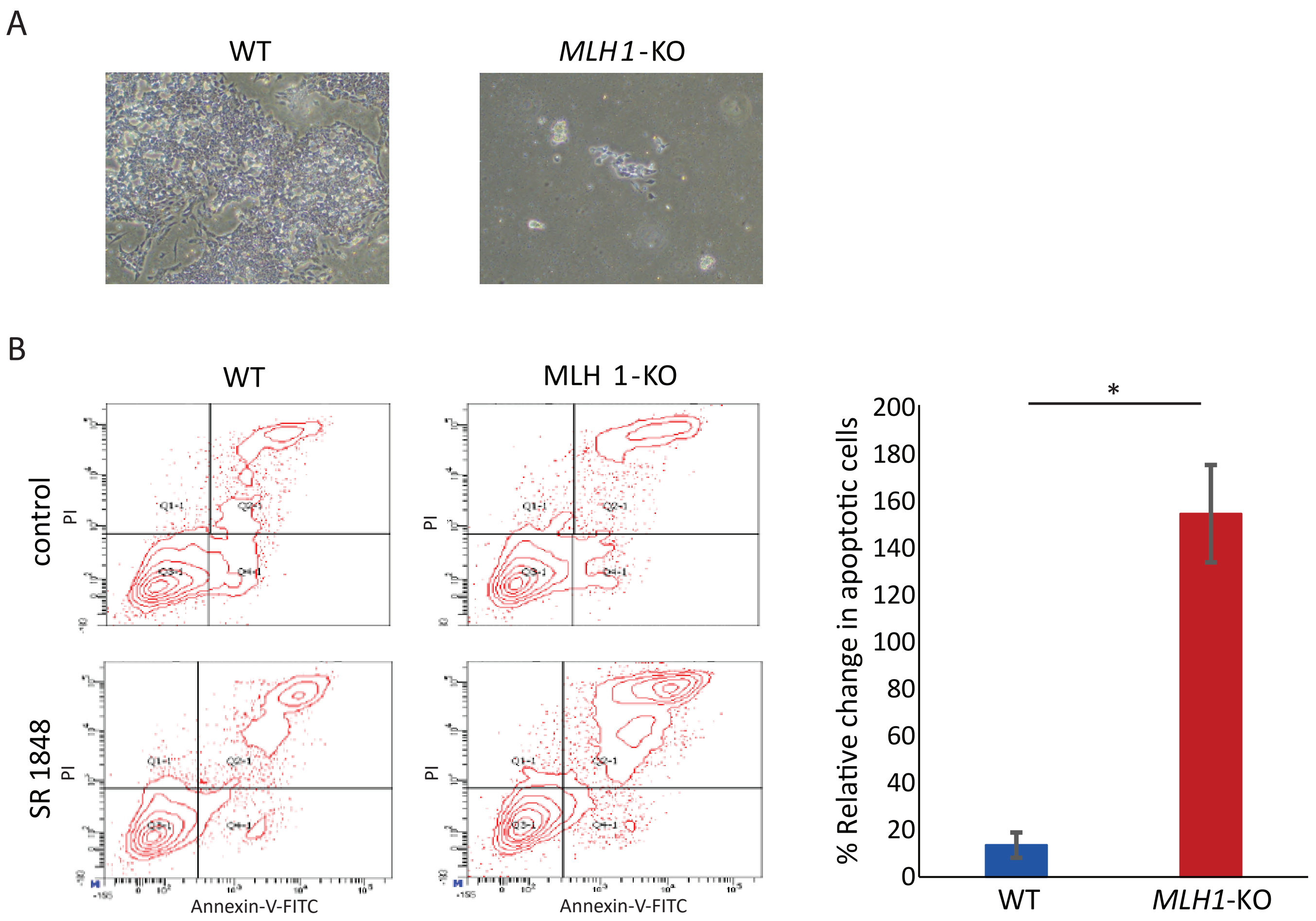

Supplementary Figure 3: Chemical validation of synthetic lethality candidate genes in haploid hESCs *MLH1* deficient model.

A. Images depicting SR1848 treated WT or *MLH1*-KO cells.

B. Annexin-V-PI staining, utilized for apoptosis detection, is depicted in the FACS density-contour plot (left) of WT/*MLH1*-KO cells treated with SR1848. quantitative analysis of apoptotic cells provided on the right (n=3, *p-value < 0.05, two-sided T-test).

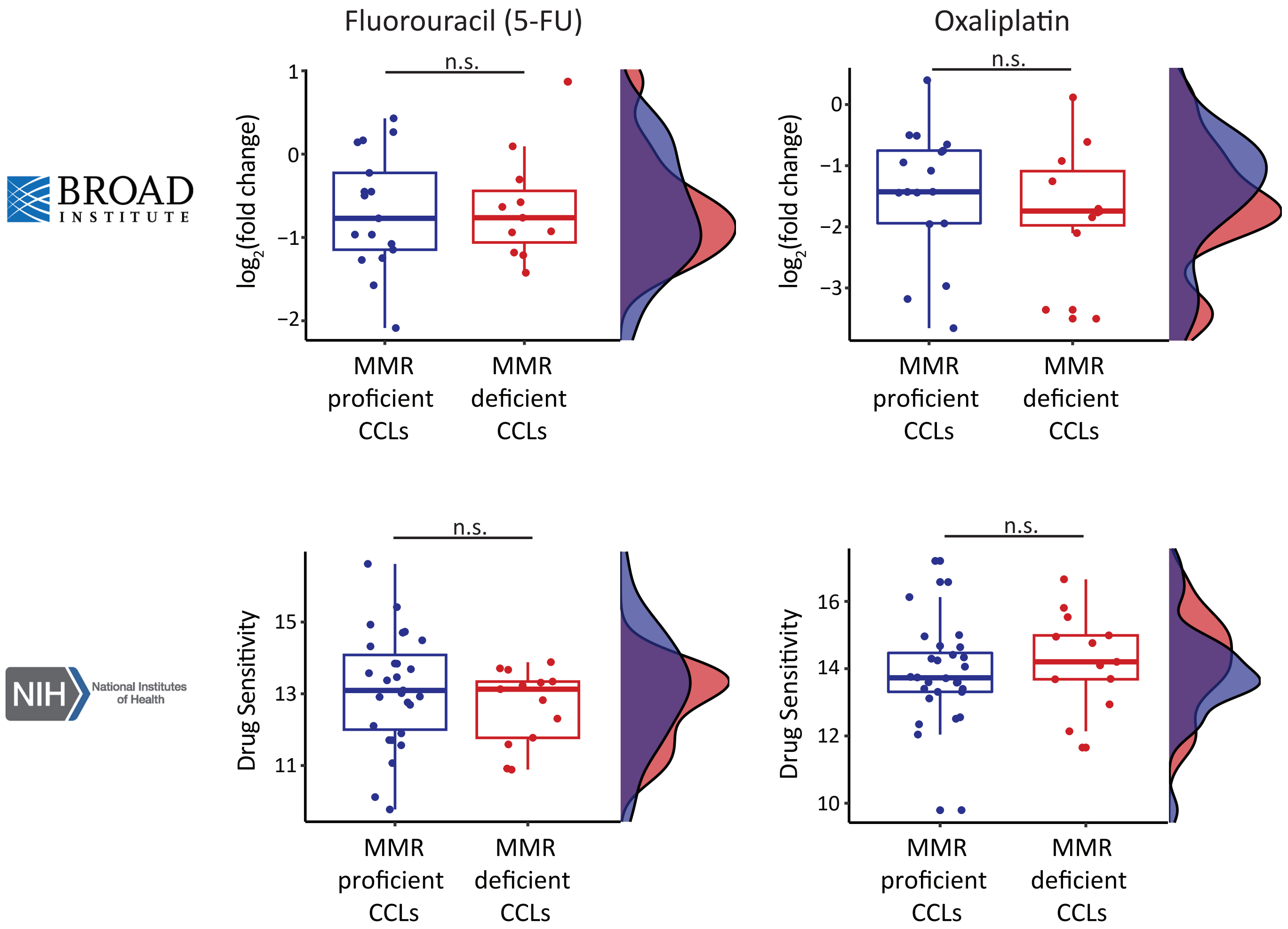

Supplementary Figure 4: Bioinformatic validation of synthetic lethality candidate genes in *MLH1* deficient CCL models.

Bioinformatic analysis of CCLs (CCLs) response to 5-FU (left) or oxaliplatin (right) treatment is presented through box plots and density plots. Shown are box plot representations, with median as center line, and 25% and 75% as box limits. MMR-proficient CCLs are represented in blue, while MMR-deficient CCLs are in red. The top panel displays sensitivity data obtained from the Broad Repurposing Library and the PRISM multiplexed cell-line viability assay. The y-axis indicates the log_2 ratio of the abundance of cells in the treatment group versus cells in the control DMSO group. The bottom panel reflects sensitivity data from the CTD2 database. All comparisons yielded non-significant results (p-value > 0.1). n.s.: not significant.

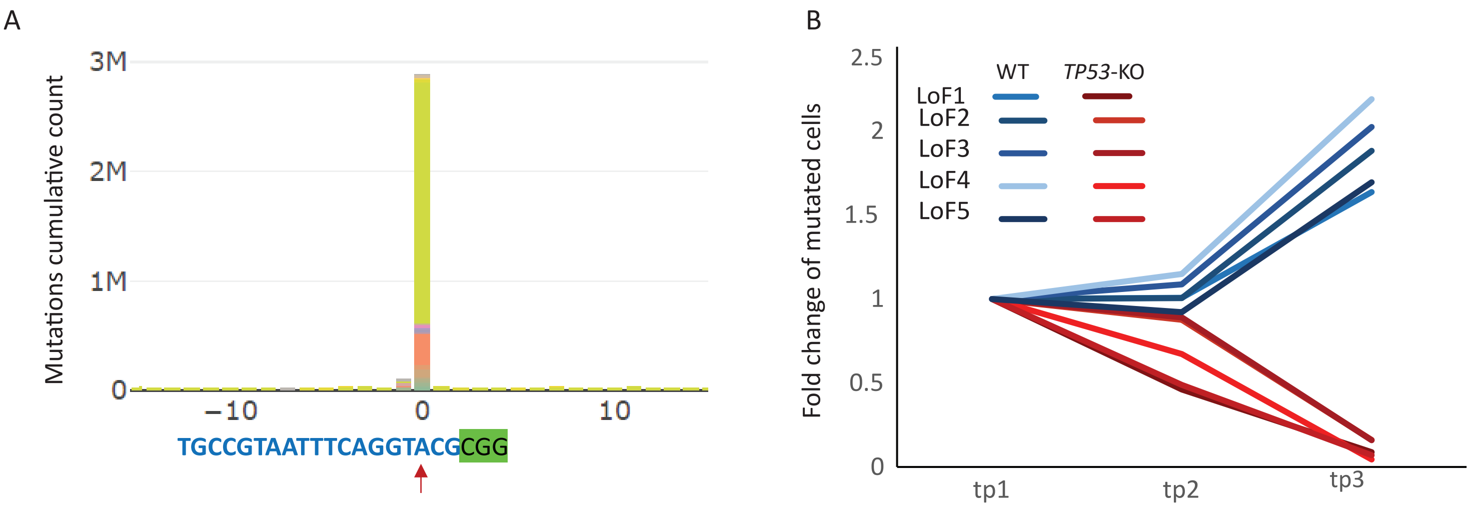

C

Pathway	Description	Enriched/depleted in $\frac{TP53\_PAB}{TP53}$	Enriched/depleted in $\frac{WT\_PAB}{WT}$
Androgen response	Genes defining response to androgens	Enriched	Depleted
KRAS signaling up	Genes up-regulated by KRAS activation	Enriched	Depleted
Hormonal pathway	Genes involved in hormone biosynthesis, degradation, regulation and signal transduction	Enriched	-
Inflammatory response	Genes defining inflammatory response	Enriched	-
TGF-BETA signaling	Genes up-regulated in response to TGFB1	Enriched	-
PPAR signaling pathway	PPAR signaling pathway	Enriched	-
KRAS signaling down	Genes down-regulated by KRAS activation	Depleted	Enriched
Insulin signaling pathway	Insulin signaling pathway	Depleted	-
Axon guidance	Axon guidance	Depleted	Enriched

Supplementary Figure 5: Genetic validation of the synthetic lethality candidate gene *MYH10*, in haploid hESCs *TP53* deficient model.

A. Location of all mutations in *MYH10* in the genetic validation. On the X-axis, we denote the mutation locations relative to the PAM sequence, where zero signifies the position of the CRISPR-Cas9 cut site, also indicated by the arrow. The Y-axis is the cumulative counts of all mutations that occurred in a specific location across all samples. for indels longer than 1, the location was defined as the one nearest to the cut site locus.

B. Top 5 LoF mutations in *MYH10* in the genetic validation. Presented mutations were selected based on summed frequency across all samples, and their differences were compared in the trends over time between the WT and *TP53*-KO. The Y-axis is the normalized values according to the first tp, that is, the first tp is equal to 1, and the other tps are the fold-change that is relative to tp 1.

C. Results of pathway enrichment analysis of WT and *TP53*-KO haploid hESCs treated or untreated with paraamino-blebbistatin. Significant pathways enriched or depleted in *TP53*-KO cells.

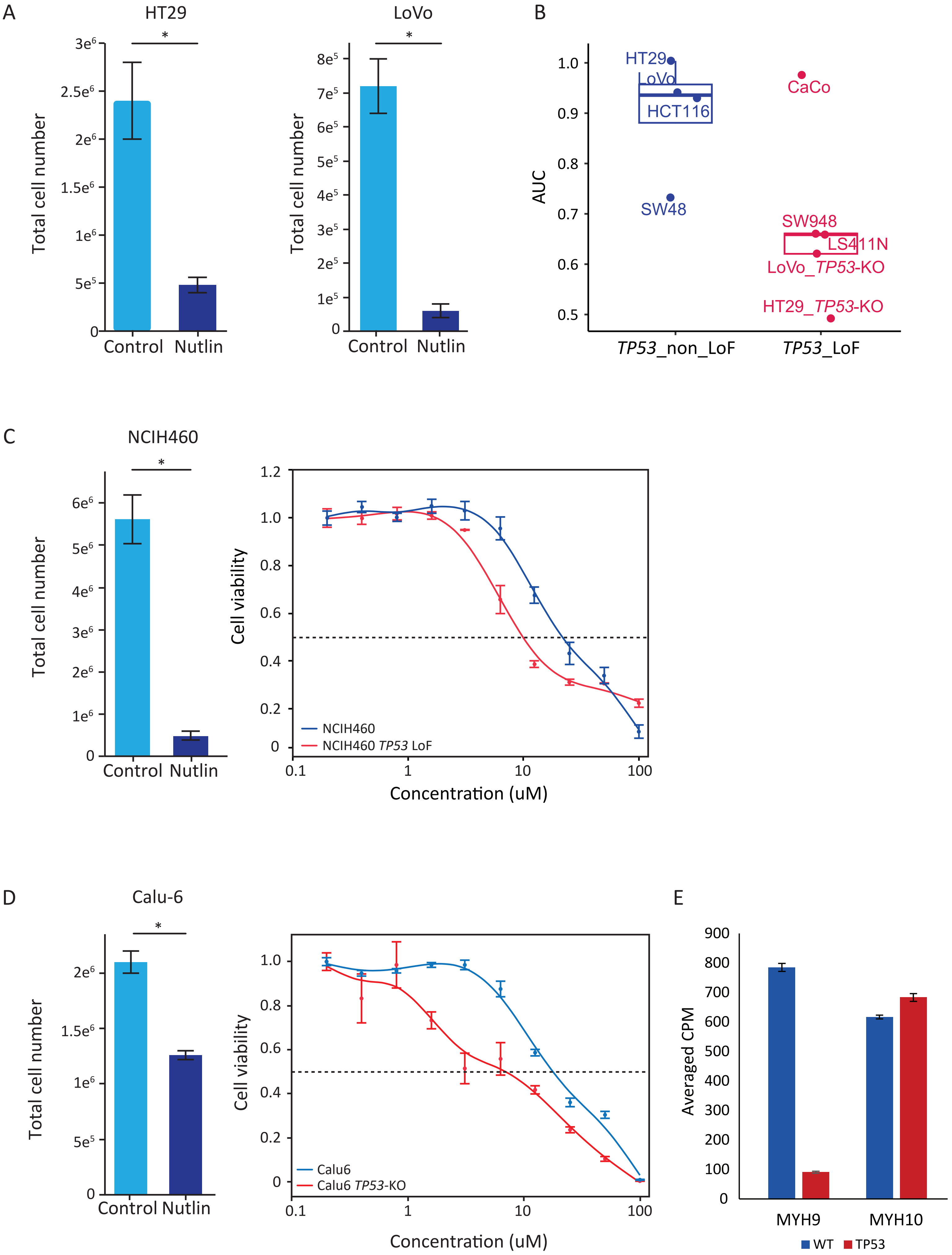
A
HT29
*
3e6
2.5e6
2e6
1.5e6
1e6
5e5
0
Total cell number
Control
Nutlin
LoVo
*
8e5
7e5
6e5
5e5
4e5
3e5
2e5
1e5
0
Total cell number
Control
Nutlin
B
AUC
1.0
0.9
0.8
0.7
0.6
0.5
HT29
LoVo
HCT116
SW48
CaCo
SW948
LS411N
LoVo_TP53-KO
HT29_TP53-KO
TP53_non_LoF
TP53_LoF
C
NCIH460
*
6e6
5e6
4e6
3e6
2e6
1e6
0
Total cell number
Control
Nutlin
1.2
1.0
0.8
0.6
0.4
0.2
0
Cell viability
NCIH460
NCIH460 TP53 LoF
0.1
1
10
100
Concentration (uM)
D
Calu-6
*
2e6
1.5e6
1e6
5e5
0
Total cell number
Control
Nutlin
1.0
0.8
0.6
0.4
0.2
0
Cell viability
Calu6
Calu6 TP53-KO
0.1
1
10
100
Concentration (uM)
E
900
800
700
600
500
400
300
200
100
0
Averaged CPM
MYH9
MYH10
WT
TP53

Supplementary Figure 6: Genetic and chemical validation of the synthetic lethality candidate gene *MYH10*, in *TP53* deficient CCLs.

A. Effect of nutlin-3 on CCLs. HT29 and LoVo are colorectal CCLs without *TP53* LoF mutation. Total number of cells was compared between treated (dark blue) and untreated (light blue) cells. X axis = sample, Y axis = total cell number.

B. Effect of blebbistatin on colorectal CCLs is presented via area under the curve (AUC) comparison. CCLs with *TP53* LoF (red) and CLLs without *TP53* LoF (Blue) were treated. Shown is box plot representation, with median as center line, and 25% and 75% as box limits X axis = CCLs, Y axis = AUC values.

C-D. Effect of nutlin-3 and blebbistatin on lung CCLs. NCIH460 (C) and Calu-6 (D) are lung CCLs originally without *TP53* LoF mutation are sensitive to nutlin-3 treatment (light blue vs. Dark blue). The CCLs underwent *TP53* knockout using CRISPR-Cas9 technology to generate isogenic cell lines. Dose-response curves of *TP53*-KO (red) and *TP53*-WT (blue) CCLs treated with blebbistatin for 3-6 days are depicted. The dots represent the mean of technical triplicates, with error bars indicating the standard error of the mean (SEM) for technical triplicates. X axis = concentration (uM), Y axis = cell viability. * = p-value < 0.05.

E. Effect of *TP53*-KO on *MYH9* and *MYH10* expression levels. Bars represent the averaged CPM of *MYH9* and *MYH10* in WT (blue bars) and *TP53*-KO (red bars) cells (n=3 for all groups).

Supplementary Table 1. List of all genes and their sgRNAs used for the custom CRISPR library.

Supplementary Table 2. sgRNA sequences used for the generation of KO cell lines.

Supplementary Table 3. List of CCLs and their *TP53* status and MMR status used for validation.

Supplementary Table 4. List of Inhibitors concentrations.
